# Self-Assembly of Asymmetrically Functionalized Titania Nanoparticles into Nanoshells

**DOI:** 10.3390/ma13214856

**Published:** 2020-10-29

**Authors:** Fredric G. Svensson, Gulaim A. Seisenbaeva, Nicholas A. Kotov, Vadim G. Kessler

**Affiliations:** 1 Department of Molecular Sciences, Swedish University of Agricultural Sciences (SLU), Box 7015, 750 07 Uppsala, Sweden; fredric.svensson@slu.se (F.G.S.); gulaim.seisenbaeva@slu.se (G.A.S.); kotov@umich.edu (N.A.K.); 2 Department of Chemical Engineering, University of Michigan, Ann Arbor, MI 48109, USA; 3 Biointerfaces Institute University of Michigan, Ann Arbor, MI 48109, USA

**Keywords:** self-assembly, nanoshells (hollow spheres), janus particles, pickering emulsion

## Abstract

Titania (anatase) nanoparticles were anisotropically functionalized in water-toluene Pickering emulsions to self-assemble into nanoshells with diameters from 500 nm to 3 μm as candidates for encapsulation of drugs and other compounds. The water-phase contained a hydrophilic ligand, glucose-6-phosphate, while the toluene-phase contained a hydrophobic ligand, *n*-dodecylphosphonic acid. The addition of a dilute sodium alginate suspension that provided electrostatic charge was essential for the self-limited assembly of the nanoshells. The self-assembled spheres were characterized by scanning electron microscopy, elemental mapping, and atomic force microscopy. Drug release studies using tetracycline suggest a rapid release dominated by surface desorption.

## 1. Introduction

Self-assembly of inorganic nanoparticles (NPs) has emerged as a promising pathway for engineering complex nano- and microscale structures [1,2], and understanding various natural phenomena [3]. Increasing sophisticated self-assembled nanostructured constructs, including lamellar titania sheets [4], snowflake-like structures [5], and hedgehog-particles [6] can be produced under mild conditions and *en mass*. Although it was believed before that NPs used for self-assembly are required to be homogenous in size and shape [7] to form complex objects, the latest studies indicate that polydispersed NPs can produce amazingly complex assemblies competing and/or exceeding those observed in the biological world [8]. In fact, monodispersed NPs with isotropic (i.e., spherical) morphology may not be the best ‘building blocks’ for engineering geometrically complex and dynamic superstructures in dispersion (rather than in solid crystals) under the conditions when a system display the thermodynamic preference to produce high-mass disorganized aggregates and has no assembly restriction related to repulsive interactions or NP symmetry. This fundamental problem can be mitigated by engineering intrinsic anisotropy in the inter-particle interactions. It can be achieved, for instance, by attaching stabilizing ligands to the particle surfaces, producing so-called Janus particles, where opposite hemispheres have opposite properties (e.g., polarity). Colloidal spheres with two or more chemical zones have attracted recent interest as anisotropic building blocks for self-assembled systems [9,10,11,12]. One way to obtain Janus particles is to partially cover a part of the particle surface and then functionalize the exposed surface with ligands to obtain an opposite polarity to the particle surface [13,14]. While the ability of NPs to act as Pickering stabilizers in an emulsion is known [15], this property has been underexploited. As the NPs migrate to the phase-boundary of two immiscible liquids to lower the interfacial energy, their surfaces will be exposed to the ligands added to the two different phases [16,17]. An area of active research involves encapsulation of biomolecules, e.g., drugs, antibodies, enzymes [18], and even living cells [19,20]. Aqueous titania sols have recently been applied to encapsulate both bacteria [21] and human immune cells with retained biological activity and good viability upon release [22]. The encapsulation of bacteria is based on deposition of particles to cover the bacterial surface, and is, therefore, not suitable for encapsulation of soluble, low-weight molecules. Rather, an emulsion route could be more efficient for capture and encapsulation of such compounds.

Titania, in both anatase and rutile phases, has shown low cell toxicity that mostly appeared to be dependent on its photocatalytic activity by production of reactive oxygen species [23]. However, titania has recently been demonstrated to induce thrombosis in contact with blood even without exposure to light [24], indicative of the incomplete understanding of biological effects of titania nanostructures. Combined with their promising chemical and biological characteristics, titania-based self-assembled superstructures can be promising for a variety of biomedical applications.

Several approaches to obtain hollow titania spheres (i.e., nanoshells) have been reported in the literature. Pang and co-workers [25] produced hollow, spherical self-assembled titania spheres by a simple hydrothermal treatment, showing promise for utilization in dye-sensitized solar cells. Seisenbaeva et al. [26] hydrolyzed molecular titanium precursors to obtain hierarchical, highly porous, spheres of titania with possible application for drug delivery. In another study, Chen and co-workers [27] utilized the Pickering emulsion effect from particles in a two-phase system. Commercial titania suspended in water was added to a n-hexadecane solution of styrene, divinylbenzene, and 2,2′-azobis (2,4-dimethyl valeronitrile). Titania nanoshells formed upon vigorous stirring and subsequently stabilized by polymerization of the organic additives at elevated temperature. A number of publications have reported self-assembled silica nanoshells, ranging from nanometer size to micrometer size [28,29]. Using the Stöber process, better particle homogeneity and size control can be achieved in the synthesis of silica NPs compared to titania NPs. This, in turn, facilitates the control over properties of the self-assembled structures and interparticle forces.

A facile method for production of anisotropically functionalized gold NPs was reported by Andala and co-workers [17]. They suspended dodecylamine-stabilized gold NPs in a water-toluene system containing hydrophobic and hydrophilic ligands with thiol functional groups. Under vigorous stirring, an emulsion formed and the Au particles acted as Pickering stabilizers by migrating to the phase-boundary between the water and toluene phases. This resulted in the formation of Janus particles. We hypothesized that the same principle could be applied to produce nanoshells from inexpensive titania nanopowders with a high degree of polydispersity taking advantage of anisotropy-guided self-assembly.

## 2. Materials and Methods

Disodium glucose-6-phosphate (Sigma-Aldrich Sweden AB, Stockholm, Sweden) *n*-dodecylphosphonic acid (Sigma-Aldrich Sweden AB, Stockholm, Sweden), disodium alginate (Fisher Scientific Gtf AB, Göteborg, Sweden), toluene (Merck AB, Solna, Sweden), tetracycline HCl (Sigma-Aldrich Sweden AB, Stockholm, Sweden) hydrothermally synthesized titania, and deionized water (DI H_2_O) were used. For characterization, a Hitachi TM-1000 (Hitachi Hightech Europe AB, Solna, Sweden) and a Hitachi FlexSEM 1000II (Hitachi Hightech Europe AB, Solna, Sweden) were used for scanning electron microscopy (SEM) imaging and elemental analyses. A Bruker atomic force microscope (AFM) FastSCAN with ScanAsyst (Blue Scientific Inc., St. John’s Innovation Centre, Cambridge, UK) was used for atomic force microscopy (AFM) imaging. A Malvern Zetasizer Nano, (Malvern Panalytical Ltd, Malvern, UK) was used for dynamic light scattering (DLS). Moreover, 1 mL aqueous sample in a plastic cuvette was analyzed at 20 °C in three replicates.

A Field Electron and Ion Company (FEI) Tecnai F30 ST, (Blue Scientific Inc., St. John’s Innovation Centre, Cambridge CB4 0WS, UK) with a 300 kV field emission gun was used for transmission electron microscopy (TEM). Gatan micrograph suite version 3.2 was used for analyzing the TEM data. The powder diffractogram was recorded by a Bruker D8 SMART diffractometer with APEX II charge-coupled device detector (graphite monochromator) (Blue Scientific Inc., St. John’s Innovation Centre, Cambridge, UK) using k(Mo-Ka) = 0.71073 Å radiation. The hydrothermal titania was synthesized as follows: 1 mL titanium (IV) ethoxide in a Teflon container was mixed with 20 μL 0.3 mM NH_4_F and, subsequently, 0.48 mL DI H_2_O was added. The Teflon container was placed in a steel autoclave and treated with the following temperature program: 15 min ramp from room temperature to 50 °C (held 30 min), then ramped at 1.6 °C min^−1^ to 100 °C (held 18 h), ramped at 1.3 °C min^−1^ to 180 °C (held 24 h), followed by a natural cool down. The obtained powder was repeatedly washed and centrifuged two times with 70% ethanol (Solveco, Rosersberg, Sweden), two times with DI H_2_O, and finally, two times with acetone (99%, Sigma-Aldrich Sweden AB, Stockholm, Sweden) following drying at room temperature. The titania nanopowder was characterized by powder X-ray diffraction and transmission electron microscopy.

**Figure A1:**
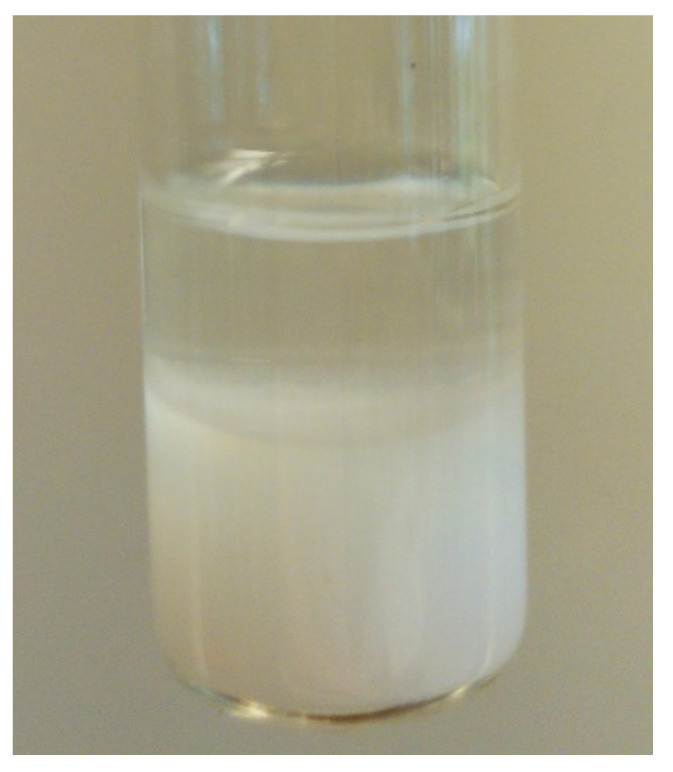
The material after stirring.

In a typical procedure, ca. 2 mg of titania was dispersed in 2 mL of toluene with 2 mM *n*-dodecylphosphonic acid by sonication for 1 h. The glass tubes were about 2 cm in diameter and the stirring bars were 15 mm × 5 mm. Immediately after sonication, the magnetic stirring was started. Under vigorous stirring, 1 mL of 0.1 w% sodium alginate aqueous solution and 2 mL of 1.6 mM glucose-6-phosphate was added simultaneously. The stirring was continued for two hours, following removal of the stirrer and the system was left to settle. The titania usually migrated to the aqueous phase to give a white foamy appearance (Figure A1). About 20 μL was pipetted to a carbon tape coated sample holder for SEM and AFM imaging. Tetracycline was chosen as a model drug for release studies. The release was followed using a Thermo Scientific GENESYS 20 UV-vis single-beam spectrophotometer (Thermo Fisher Scientific AB, Uppsala, Sweden), measuring at 400 nm. A standard series of tetracycline dissolved in 0.9% aqueous sodium chloride was used to calculate the release through the Lambert-Beer law; a = ε c l. A linear relationship (R^2^ = 0.996) was obtained for the standard series.

## 3. Results and Discussion

**Figure A2:**
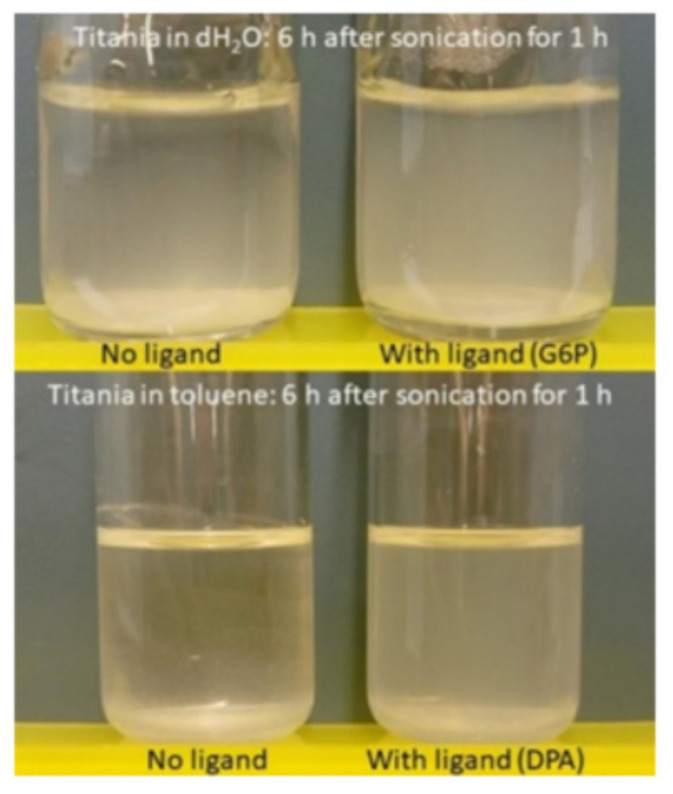
Titania sonicated in water or toluene compared with titania sonicated in the presence of ligands. The addition of ligands stabilizes the particles and less precipitation has occurred six hours after sonication.

As ligands for functionalization, glucose-6-phosphate (G6P, hydrophilic) and *n*-dodecylphosphonic acid (DPA, hydrophobic) were chosen. Both the phosphate and phosphonate functions have documented affinity for titania surfaces [30,31], even at lowered pH [32]. In addition, their presence is easily confirmed from phosphorous by elemental analysis compared to the carboxylate function. Titania NPs formed in the sol-gel process have size distribution in 3–5 nm window of diameters [21,26,33]. They tend to form large aggregates due to their high surface energy and sonication was used to reduce the size of the aggregates. Sonication of titania powders in pure solvents (toluene or water) apparently lead to fast re-aggregation. Sonication together with either DPA or G6P was found to markedly stabilize the particles in solution (Figure A2). Further, to stabilize the self-assembled structures, alginate, an anionic polysaccharide, was added. Alginate may interact with G6P via hydrogen bonding, and possibly also electrostatically with the titania surface, as well as increasing solution viscosity.

**Figure 1:**
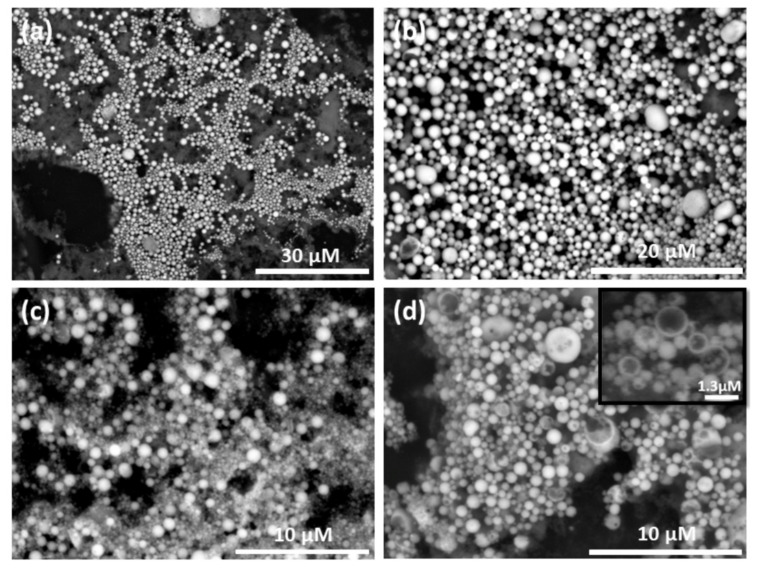
Scanning electron micrographs of self-assembled titania nanoshells. (**a**) Lower magnification micrograph of nanoshells dispersed over a matrice. (**b**) Micrograph of titania nanoshells of rather narrow size distribution. Average diameter calculated from 40 random nanoshells was ca. 1 μm (standard deviation (SD) 760 nm). (**c**) An example of nanoshells with higher size distribution. (**d**) Micrograph with several broken nanoshells. Average diameter calculated from 40 random nanoshells was ca. 680 nm (SD = 410 nm). The inset in (**d**) shows several incomplete nanoshells at 9000× magnification.

**Figure A3:**
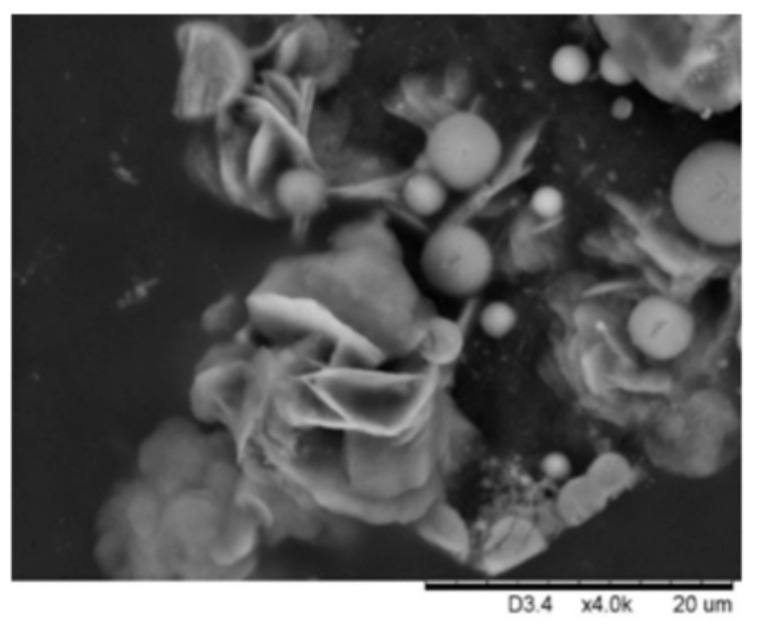
Competition between assembly of nanoshells and sheets when higher amounts of titania and alginate were used.

When increasing the amounts of titania and alginate, a competition between formation of spheres and sheets was observed (Figure A3). By vigorously stirring a small amount of titania in a system of G6P (in DI H_2_O), DPA (in toluene), and dilute alginate; hollow, self-assembled titania spheres were obtained (Figure 1).

**Figure 2:**
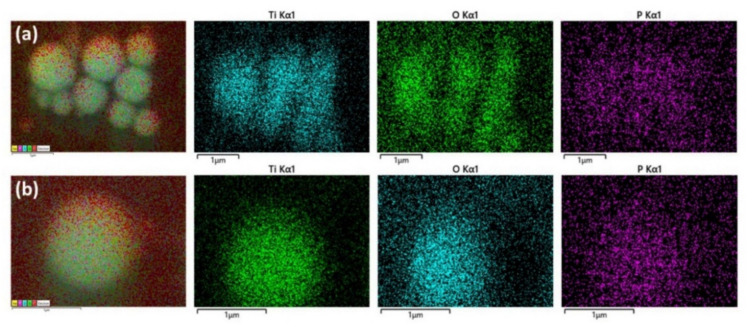
Elemental mapping of self-assembled nanoshells, showing the presence of titanium, oxygen, and phosphorous. Analysis of (**a**) an aggregate of self-assembled hollow spheres and (**b**) an individual hollow sphere.

Elemental mapping confirms the presence of titanium, oxygen, and phosphorus in the spheres, Figure 2.

Carbon is present on the spheres (not shown), but in lower amounts compared to the surrounding carbon tape. The relatively high presence of phosphorous on the spheres, together with no formation of sphere in the absence of the organic ligands, indicates that they are bond to the TiO_2_ NPs and are essential for self-assembly process. The spheres are dispersed on a thin layer of non-assembled materials (Figure 1a), which contributes to diffuse signals in the elemental analysis in Figure 2, particularly for phosphorus. The hollow volume of spheres, and the amount of broken/incomplete spheres, varies between batches. When dried, the spheres survive for several days on the sample holder carbon tape. This would indicate stability of the structures after presumed evaporation of encapsulated toluene. They were observed to be stable in dispersion for at least one month, as determined by SEM imaging.

**Figure A4:**
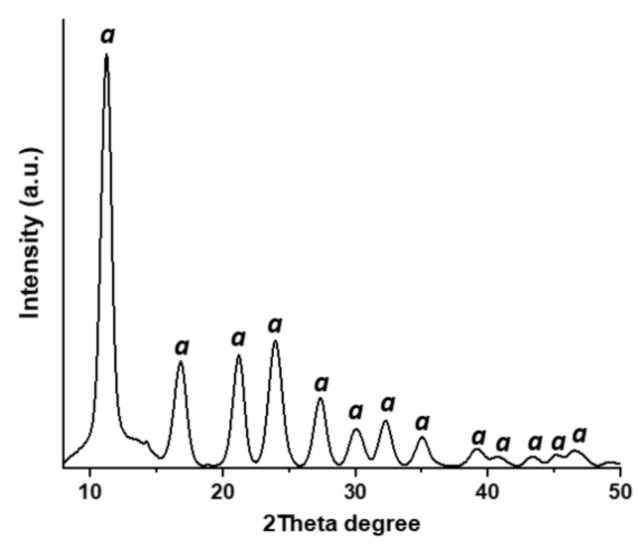
PXRD pattern of the hydrothermally synthesized titania. Peaks labeled “*a*” belongs to the anatase phase.

**Figure A5:**
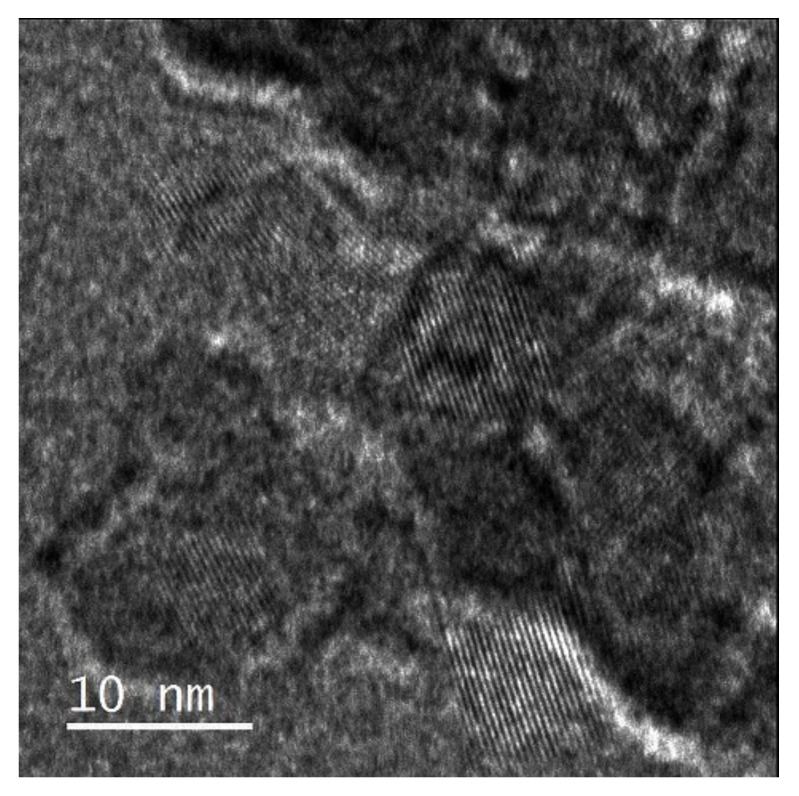
TEM micrograph of hydrothermally synthesized titania.

The hydrothermally synthesized titania NPs was nanocrystalline in the anatase phase, as determined by powder X-ray diffraction (Figure A4). Transmission electron microscopy (TEM) micrographs revealed primary anatase particles of about 8 nm (Figure A5). Attempts with highly crystalline anatase powders (annealed >500 °C) and different titanate perovskites from solid-state syntheses failed to produce nanoshells. NP growth and increased polydispersity during high annealing temperatures is hypothesized, and have a negative impact on the self-assembly process.

**Figure 3:**
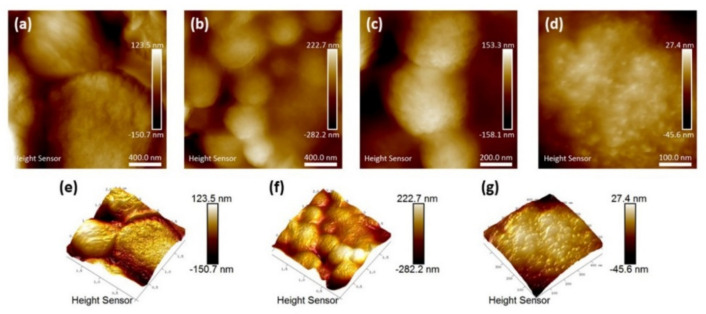
Atomic force micrographs of titania nanoshells. (**a**) Micrographs of a few larger nanoshells. (**b**) An aggregate of smaller nanoshells average diameter is 420 nm (SD = 80 nm). (**c**) Magnified micrograph of three small nanoshells average diameter is 416 nm (SD = 55 nm). (**d**) Magnified micrograph of the surface of a nanoshell. Nanoshell topography is shown in (**e**), and (**f**), and a three-dimensional (3D) image of a surface in (**g**).

To obtain more information about structural features of the surface of the spheres, atomic force microscopy (AFM) was used. It can clearly be seen that the spheres are built up by smaller aggregates and they have porous surfaces (Figure 3).

Their spherical structure is visualized in greater detail in the AFM three-dimensional (3D) images (Figure 3e,f). The building blocks are presumably functionalized secondary particles, rather than primary NPs. The outer diameter of the nanoshells are usually within 0.5 μm to 3 μm, with an average size of approximately 1 μm, which is much smaller compared to those reported by Chen et al. [27] of about 20–50 μm. The hydrodynamic size in solution was estimated by dynamic light scattering (DLS). An average particle size of 853 nm (SD 57 nm, polydispersity index 0.451) for three replicate runs were found, which is in agreement with the observations from SEM and AFM. However, because of the wide size range (500 nm to 3 μm) of the nanoshells, the polydispersity index was relatively high. It should be noted that DLS is most accurate for particle sizes below 300 nm and the measured average should be seen as the average of a wider size distribution.

**Figure 4:**
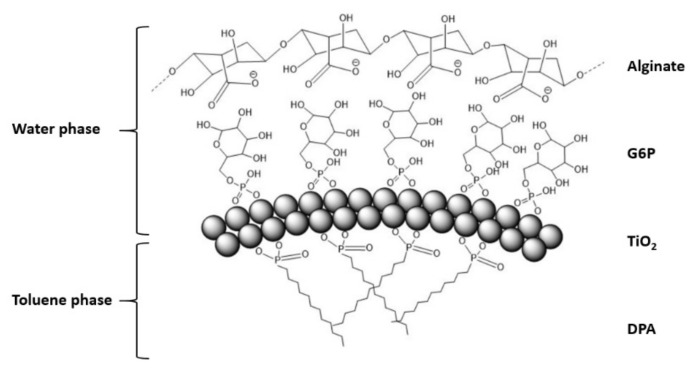
Proposed structure of self-assembled nanoshells. The inner side of the spheres are coated with the hydrophobic ligand (DPA), facing encapsulated toluene, and the outer side is coated with the hydrophilic ligand (G6P), facing the water phase. The alginate polymer helps stabilizing the nanoshells and may interact with the hydrophilic ligand via hydrogen bonding, and potentially via electrostatic interaction with the titania surface.

Vigorous mixing of the titania in the two-phase system results in a white, foamy aqueous phase after settling. This suggests the spheres have their outer surface coated with the hydrophilic G6P ligand, while the DPA ligands may reside on the inside. Their production without the addition of a dilute alginate suspension was not successful. The stabilizing effect may be a result of hydrogen bonding between G6P and alginate. A plausible mechanism for their self-organization is presented in Figure 4.

The preferential assembly of Janus NPs into nanoshells is a direct analog of self-assembly of amphiphilic surfactants in micelles because packing of the hydrophobic tails on NPs reduces their Gibbs free energy in aqueous media. The alginate macromolecules adsorbing onto the NPs and nanoshells is expected to increase the electrostatic repulsion between the constituent particles required for the self-limited self-assembly process [34,35].

## 4. Drug Release

**Figure 5:**
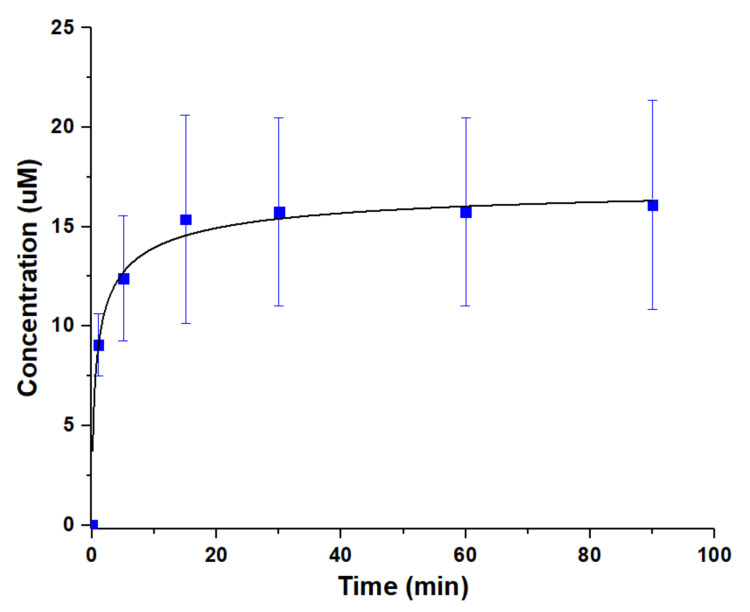
Release profile from the material for tetracycline in physiological sodium chloride at room temperature. Error bars are standard deviation.

**Figure A6:**
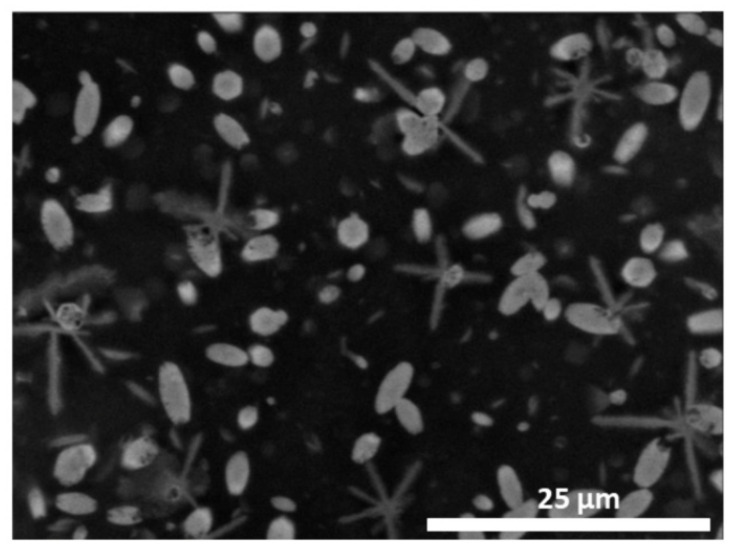
Change in morphology from spherical assemblies to oval assemblies at high loadings of drugs (2 mM). Star shaped assemblies are also emerging, mostly consisting of titanium, sodium and phosphorous according to EDS. These structures are presumably formed through evaporation induced self-assembly (EISA) during drying on the carbon tape (on SEM sample holder) from suspended NPs and sodium ions directed by the organic ligands.

Drug release from the material were investigated to identify the potential venues of the future development of the self-assembled titania nanoshells. The antibiotic tetracycline was added to the aqueous phase during mixing. It was noticed that the spherical shape turned oval when attempting to encapsulate different bioactive compounds (Figure A6). The yield of spheres also decreased, which may be explained by an emulsifying of the drugs, disturbing the interfacial tension between water and toluene. Some material was dried on glass slides and drug release was studied in 0.9% sodium chloride aqueous solution under slow magnetic stirring at room temperature. In an attempt to determine the drug loading capacity, nanoshells were dissolved in 0.1 M citric acid within 24 h. The loading capacity was estimated to ca. 1.8 mmol/g (SD 0.17∙mmol/g) for tetracycline. Release was followed spectrophotometrically. A rather quick release take place during the first 15 min followed by a plateau (Figure 5).

This suggests release from weakly interacting surface adsorbed drug molecules accompanied by the dynamic hydrogen-bonding interactions with the alginate layer on the nanoshells. Similar rapid release profiles of weakly interacting drugs have been reported by Evdokimova et al. [36], from triclosan adsorption to pure nanocellulose, and by Kulak and co-workers [37], who studied the release of ibuprofen from porous inorganic microparticles.

## 5. Conclusions

Herein we report a proof of principle for the self-assembly of titania nanoshells from anisotropically functionalized titania NPs. The asymmetry of the NPs was imparted by taking advantage of their ability to stabilize Pickering emulsion step, enabling anisotropically functionalized titania NPs. Addition of alginate was found to be essential for the successful formation of the nanoshells guiding the self-assembly of polydispersed NPs away from the high-mass disorganized agglomerates toward self-limited nanoshells that displayed relatively high monodispersity. Drug release kinetics of tetracycline indicates the successful utilization of the nanoshells for drug delivery. The release profile suggest a surface adsorption of drugs with fast release. 

Future work would need to be focused on increasing the nanoshell yield and their efficient separation from precursors and nanosheets. Other anisotropy-guided self-assembly could be investigated, e.g., more hydrophobic and bulkier hydrocarbon chains. Addition of bioactive compounds (which could act as emulsifiers) have an effect on the self-assembly process, and this needs optimization for potential use for encapsulation, and other applications benefiting from the simplicity and universality of the process.
